# Simultaneous Activation of Iron- and Thiol-Based Sensor-Regulator Systems by Redox-Active Compounds

**DOI:** 10.3389/fmicb.2017.00139

**Published:** 2017-02-02

**Authors:** Kang-Lok Lee, Ji-Sun Yoo, Gyeong-Seok Oh, Atul K. Singh, Jung-Hye Roe

**Affiliations:** School of Biological Sciences and Institute of Microbiology, Seoul National UniversitySeoul, South Korea

**Keywords:** redox-sensitive regulators, Fe–S cluster, cysteine thiol, reactive electrophiles, quinones, actinobacteria

## Abstract

Bacteria in natural habitats are exposed to myriad redox-active compounds (RACs), which include producers of reactive oxygen species (ROS) and reactive electrophile species (RES) that alkylate or oxidize thiols. RACs can induce oxidative stress in cells and activate response pathways by modulating the activity of sensitive regulators. However, the effect of a certain compound on the cell has been investigated primarily with respect to a specific regulatory pathway. Since a single compound can exert multiple chemical effects in the cell, its effect can be better understood by time-course monitoring of multiple sensitive regulatory pathways that the compound induces. We investigated the effect of representative RACs by monitoring the activity of three sensor-regulators in the model actinobacterium *Streptomyces coelicolor*; SoxR that senses reactive compounds directly through oxidation of its [2Fe–2S] cluster, CatR/PerR that senses peroxides through bound iron, and an anti-sigma factor RsrA that senses RES via disulfide formation. The time course and magnitude of induction of their target transcripts were monitored to predict the chemical activities of each compound in *S. coelicolor*. Phenazine methosulfate (PMS) was found to be an effective RAC that directly activated SoxR and an effective ROS-producer that induced CatR/PerR with little thiol-perturbing activity. *p*-Benzoquinone was an effective RAC that directly activated SoxR, with slower ROS-producing activity, and an effective RES that induced the RsrA-SigR system. Plumbagin was an effective RAC that activated SoxR, an effective ROS-producer, and a less agile but effective RES. Diamide was an RES that effectively formed disulfides and a weak RAC that activated SoxR. Monobromobimane was a moderately effective RES and a slow producer of ROS. Interestingly, benzoquinone induced the SigR system by forming adducts on cysteine thiols in RsrA, revealing a new pathway to modulate RsrA activity. Overall, this study showed that multiple chemical activities of a reactive compound can be conveniently monitored *in vivo* by examining the temporal response of multiple sensitive regulators in the cell to reveal novel activities of the chemicals.

## Introduction

Bacteria are constantly exposed to oxidative stress conditions induced by diverse redox-active chemical compounds encountered in the environment, whether in abiotic natural habitats or at the interphase with plant or animal hosts. When they penetrate cell membranes, some redox-active compounds (RACs) generate reactive oxygen species (ROS) such as superoxide anions and peroxides via redox cycling (Imlay, 2013). Other RACs directly oxidize targets in the absence of redox cycling activity (Okegbe et al., 2012). Reactive electrophile species (RES) form disulfides or S-conjugated products on sensitive cysteine thiols of proteins and small molecules such as glutathione or its equivalents, including mycothiol and bacillithiol (Rudolph and Freeman, 2009; Jacobs and Marnett, 2010; Hillion and Antelmann, 2015).

Redox-active compounds include various phenazines and quinones produced by plants, fungi, and bacteria or synthetic derivatives administered as antibiotics or anticancer agents. Phenazines, a large group of nitrogen-containing heterocyclic compounds naturally produced by bacteria can alter cellular redox states and modify gene expression (Pierson and Pierson, 2010). Quinones are found in pigments, antibiotics, vitamin K, coenzymes, and diverse anti-cancer drugs. They can modify cellular redox states and exert cytotoxicity by reacting with thiols or amine groups and generating ROS through redox cycling (O’Brien, 1991). Benzoquinones and naphthoquinones are known to be very electrophilic and thiol-reactive, readily forming ROS through a continuous cycle of reduction and autooxidation.

Cellular effects of RACs *in vivo* vary in different organisms, depending on multiple factors. These factors include their permeability, the presence of reductases to support redox cycling, the redox potential of possible targets, and the efficiency of systems that modify, degrade, and export molecules. For example, paraquat (methyl viologen) exerts its toxic effects through redox cycling, generating superoxide anions and ROS in plants, mammals, and *Escherichia coli*, but not in the actinobacterium *Streptomyces coelicolor* (Hassan and Fridovich, 1979; Bus and Gibson, 1984; Sheplock et al., 2013; Singh et al., 2013). Menadione (2-methyl-1,4-naphthoquinone; vitamin K3) and plumbagin (5-hydroxy-2-methyl-1,4-naphthoquinone) exert their toxic effects in *Saccharomyces cerevisiae* by generating ROS at millimolar concentrations and by S-thiolation at micromolar concentrations, respectively (Castro et al., 2008). In *E. coli*, however, menadione and plumbagin generate similar amounts of ROS and toxicity at 100-micromolar concentrations (Farr and Kogoma, 1991). Therefore, generalization based on studies from a few limited model systems may be insufficient to reveal the actual cellular effects. Considering the complexity of redox reactions, examining the cellular effects of RACs in a broader range of organisms is necessary to understand these reactions.

*Streptomyces coelicolor* is an actinobacterial model organism that inhabits soil. Streptomycetes produce a diverse array of antibiotics and bio-modulatory molecules, and are equipped with equally diverse mechanisms to sense and respond to these metabolites that are produced endogenously or exogenously (Bentley et al., 2002; Hopwood, 2007). Several sensor-regulators that respond to RACs have been elucidated in *S. coelicolor* (den Hengst and Buttner, 2008). They include SoxR, which senses RACs directly through oxidation of its [2Fe–2S] cluster (Singh et al., 2013; Lee et al., 2015), OxyR and CatR that sense peroxides (Hahn et al., 2000, 2002), OhrR that senses organic peroxides (Oh et al., 2007), Rex that senses NADH/NAD+ (Brekasis and Paget, 2003), [4Fe–4S]-containing NsrR that senses nitric oxide (Tucker et al., 2008; Crack et al., 2015), and the Zn-containing anti-sigma factor RsrA that senses RES through zinc-coordinating cysteine residues (Kang et al., 1999; Park and Roe, 2008; Rajasekar et al., 2016). In response to each triggering compound, these sensor-regulators induce a few to 100s of genes to defend cells against the damaging effects of the compounds. For example, activated (oxidized) SoxR induces several genes that may function in export and redox modifications (Dietrich et al., 2008; Dela Cruz et al., 2010; Shin et al., 2011; Naseer et al., 2014). Oxidized CatR, an ortholog of PerR from *Bacillus subtilis* (Lee and Helmann, 2006), derepresses the *catA* gene encoding a catalase (Hahn et al., 2000). Upon oxidation, RsrA dissociates from its binding partner SigR (Kang et al., 1999), which then induces more than 100 direct target genes related with thiol reduction, thiol synthesis, recycling of the small thiol molecule mycothiol (MSH), and protein quality control (Kim et al., 2012). Mycothiol, the functional equivalent of glutathione in actinobacteria as bacillithiol in firmicutes, maintains thiol redox homeostasis and detoxifies reactive electrophiles (Newton et al., 2008; Park and Roe, 2008; Gaballa et al., 2010).

In this study, the intracellular effects of several redox-perturbing chemicals were monitored by assessing the activity of three representative redox-sensitive regulators in *S. coelicolor*: SoxR, CatR, and RsrA. The [2Fe–2S] of SoxR in *S. coelicolor* (ScSoxR) is not oxidized by superoxide, but is directly oxidized by some RACs (Singh et al., 2013). In aerobically grown *E. coli*, where paraquat can generate superoxide anion radical, heterologously expressed SoxR from *S. coelicolor* (ScSoxR) is not activated (Singh et al., 2013). SoxR from *E. coli* (EcSoxR) can be directly oxidized by RACs such as paraquat under anaerobic condition or inside *S. coelicolor* cells where paraquat does not produce superoxide (Gu and Imlay, 2011; Singh et al., 2013). EcSoxR can be oxidized by superoxide as well (Fujikawa et al., 2016). Therefore, ScSoxR is a specific sensor for [2Fe–2S]-oxidizing RACs rather than ROS, to which CatR/PerR responds. RsrA is known to be oxidized by diamide and by other RES that can deplete MSH pool by conjugation (Paget et al., 1998; Kang et al., 1999; Park and Roe, 2008), and hence is a suitable sensor for thiol perturbation. Disulfide bond formation between conserved zinc-coordinating cysteines has been shown to inactivate RsrA (Li et al., 2003; Bae et al., 2004; Rajasekar et al., 2016). Whether, RsrA is directly alkylated by RES has not been shown. The time-course of induction of their target gene transcripts was monitored to obtain insights into the redox signals that these compounds may generate in cells.

## Materials and Methods

### Strains, Plasmids, Chemicals, and Growth Conditions

Spores of *S. coelicolor* A3(2) strain M145 were inoculated into YEME liquid medium containing 10% sucrose and incubated at 30°C (Kieser et al., 2000). γ-Actinorhodin was isolated from a plate culture of *S. coelicolor* M145 cells on R2YE as described previously (Shin et al., 2011). *E. coli* cells were grown in Luria-Bertani (LB) medium at 37°C. Toxoflavin was kindly provided by Prof. Ingyu Hwang (College of Agricultural Life Sciences, SNU). Other chemicals were obtained from Sigma-Aldrich. The chemical stock solutions were prepared fresh at 100 mM concentrations before each treatment, and used in treatments at final concentrations of actinorhodin (200 nM), pyocyanin (10 μM), toxoflavin (20 μM), phenazine methosulfate (50 μM), paraquat (200 μM), plumbagin (25–50 μM), menadione sodium bisulfite (MDs, 500 μM), menadione (MD, 350 μM), *p*-benzoquinone (50 μM), diamide (500 μM), and monobromobimane (20 μM).

### RNA Preparation and S1 Nuclease Protection Assay

*Streptomyces coelicolor* cells grown to an OD_600_ of 0.3–0.4 in YEME were treated with various chemicals for 5–120 min. Harvested cells were disrupted by sonication in Kirby mix. RNA was isolated and the S1 nuclease protection assay was performed as described previously (Kieser et al., 2000). For each assay, 50 μg RNA was used to analyze SCO2478 and *catA* transcripts, and 20 μg RNA was used to analyze *sigR* transcripts. To generate gene-specific S1 mapping probes, PCR products encompassing *sigR* (-265 to +80 nt relative to the start codon), SCO2478 (-177 to +100 nt relative to the start codon), and *catA* (-115 to +153 nt relative to the start codon) promoter regions were obtained.

### Fluorescence Measurement of Intracellular ROS

Exponentially grown *S. coelicolor* cells (OD_600_ ∼0.2–0.3) were treated with the indicated amounts of each chemical for 30 min, followed by harvest of the cells by centrifugation for 3 min at 5,000 × *g* at 4°C. Cell pellets were briefly washed twice with ice-cold P buffer [103 mg/l sucrose, 0.25 mg/l potassium sulfate, 2 ml trace element solution, 0.05 mg/l KH_2_PO_4_, 2.03 mg/l MgCl_2_⋅6H_2_O, 3.68 mg/l CaCl_2_⋅2H_2_O, and 100 ml 0.25 M TES buffer (pH 7.2)]. The trace element solution contained 40 mg/l ZnCl_2_, 200 mg/ml FeCl_3_⋅6H_2_O, 10 mg/ml CuCl_2_⋅2H_2_O, 10 mg/ml MnCl⋅4H_2_O, 10 mg/l Na_2_B_4_O_7_⋅10H_2_O, and 10 mg/l (NH_4_)_6_Mo_7_O_24_⋅4H_2_O (Kieser et al., 2000). Cells were resuspended in pre-warmed P buffer containing 5 μM DCFH_2_-DA (2′-7′-dichlorofluorescein diacetate; Sigma) and incubated for 30 min at 30°C in the dark. Intracellular conversion of DCFH_2_-DA by esterase in the cell produces non-fluorescent DCFH_2_, which can be oxidized by ROS to produce fluorescent DCF (Keston and Brandt, 1965; Bass et al., 1983). DCF fluorescence was measured in a fluorometric plate reader (EnVision Multilabel Plate Readers, Perkin Elmer) with excitation and emission at 492 and 535 nm, respectively. The optical density of each sample was measured at 600 nm in a spectrophotometric plate reader (PowerWave X, BioTek) to normalize the fluorescence by the amount of cells in each well.

### Western Blot Analysis of the Redox States of RsrA

Exponentially grown *S. coelicolor* cells (OD_600_ ∼0.3–0.4) were treated with 0.5 mM diamide or 50 μM benzoquinone for 5–60 min, followed by fixation with 10% trichloroacetic acid (TCA) for 20 min on ice. The fixed cells from 15 ml cultures were harvested and ruptured in 12.5% TCA with sonication. Precipitated proteins were washed twice with cold acetone, solubilized in TSE buffer (50 mM Tris-HCl at pH 7.5, 0.1% sodium dodecyl sulfate [SDS], 10 mM EDTA) containing 10 mM AMS (4-acetamido-4′-maleimidylstilbene-2,2′-disulfonic acid; MW 536.4), and incubated at room temperature for 2 h. Solubilized proteins were precipitated with 10% TCA, washed with acetone twice, and dissolved in 100 ml TSE buffer. Protein samples were mixed with SDS loading buffer containing 50 mM dithiothreitol (DTT) and resolved by 15% SDS-polyacrylamide electrophoresis (PAGE). Purified RsrA was run in parallel, with or without AMS treatment in the presence of DTT, to indicate the positions of fully reduced RsrA (up to 7 ASM-adducts per molecule) or oxidized RsrA, respectively. After electrophoresis, the proteins were transferred to a nitrocellulose membrane, followed by blocking with skim milk. Immuno-detection was performed using a polyclonal rabbit antibody against RsrA protein (AbClon), with the anti-rabbit IgG secondary antibody (Santacruz) diluted to 1:5000 and 1:3000, respectively, followed by enhanced chemilumenescence (ECL) detection (Amersham Life Science). To determine the amount of total RsrA in each sample, cell extracts were mixed with SDS loading buffer, followed by 15% SDS-PAGE and Western blot analysis.

### Purification of RsrA and Analysis of its Thiol-Modified Forms

His-tagged RsrA was overproduced in *E. coli* BL21(λDE3)pLys harboring pET15b-RsrA, followed by purification with an Ni-NTA column as described previously (Kang et al., 1999). After removing the His-tag with thrombin, RsrA was purified by gel filtration chromatography on a HiLoad Superdex 75 column (GE Healthcare) in TN buffer (40 mM Tris-HCl at pH 7.5, 0.2 mM NaCl). Purified proteins were quantified using Pierce BCA Protein Assay Kit (Thermo Scientific). Reduced RsrA was prepared by incubating 200 nM RsrA in 10 mM DTT at 25°C for 1 h, with the subsequent addition of 400 nM ZnSO_4_ for 1 h in an anaerobic chamber (Coy) with 5% H_2_, 5% CO_2_, and 90% N_2_. Free zinc and DTT was removed by using a PD-10 desalting column and anaerobically prepared TN buffer. To analyze para-benzoquinone (BQ)-modified RsrA, 100 nM reduced RsrA in 300 μL buffer was treated with 50 μM BQ for 10 or 20 min, and electrophoresed on SDS-PAGE followed by silver staining. For mass analysis, 50 μM reduced RsrA in 300 μL buffer was treated with 1 mM BQ for 20 min, followed by denaturation in 8 M urea.

### LC–MS/MS Analysis

Analytical capillary columns (100 cm × 75 μm i.d.) and trap columns (2 cm × 150 μm i.d) were packed in-house with 3 μm Jupiter C18 particles (Phenomenex, Torrance, CA, USA). The long analytical column was placed in a column heater (Analytical Sales and Services, Pompton Plains, NJ, USA) regulated to a temperature of 45°C. Dionex Ultimate 3000 RSLC nano-system (Thermo Scientific, Sunnyvale, CA, USA) was operated at a flow rate of 350 nL/min over 110 min with linear gradient ranging from 95% solvent A (H_2_O with 0.1% formic acid) to 40% of solvent B (acetonitrile with 0.1% formic acid). The peptide samples were analyzed on a Q-Exactive mass spectrometer (Thermo Scientific) equipped with an in-house customized nanoelectrospray ion source. Precursor ions were acquired (m/z 400–1800) at 70 K resolving power and the isolation of precursor for MS/MS analysis was performed with a 2.0 Th. Higher-energy collisional dissociation (HCD) with 25% collision energy was used for sequencing with a target value of 1e6 ions determined by automatic gain control. Resolving power for acquired MS2 spectra was set to 17,500 at m/z 200 with 60 ms maximum injection time. All MS/MS data were searched by MS-GF+ algorithm (v.9979) at 10 ppm of precursor ion mass tolerance against UniProt *S. coelicolor* DB (entry 8171, Oct 18 2016). The following search parameters were applied: semi-tryptic digestion, and dynamic BQ (delta monoisotopic mass: 108.021) of cysteine residue. The false discovery rate (FDR) was set at 1% for peptide spectrum match including unlabeled peptides resulting in zero FDR for the labeled peptides.

## Results and Discussion

### Modulation of the Activity of Redox-Sensitive Regulators in *S. coelicolor* by Redox-Active Compounds

**FIGURE 1:**
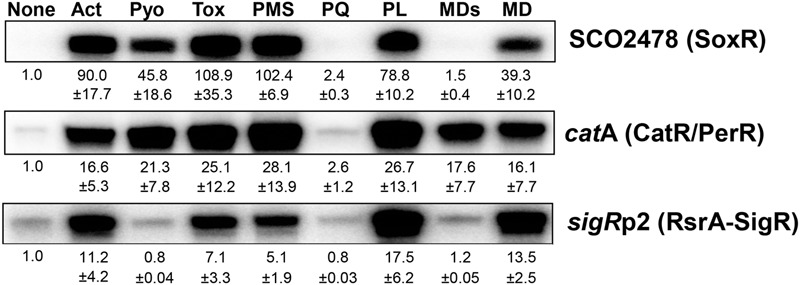
**Differential activation of redox-sensitive regulators by redox-active compounds (RACs) in *Streptomyces coelicolor*.** Exponentially grown *S. coelicolor* cells (OD_600_ 0.4–0.5) were treated with the following RACs for 30 min: actinorhodin (Act, 200 nM), pyocyanin (Pyo, 10 μM), toxoflavin (Tox, 20 μM), phenazine methosulfate (PMS, 50 μM), paraquat (PQ, 200 μM), plumbagin (PL, 25 μM), menadione sodium bisulfite (MDs, 500 μM), and menadione (MD, 350 μM). Activity modulation of SoxR, CarR/PerR, and RsrA-SigR sensor-regulators was monitored by S1 mapping of their target gene transcripts, SCO2478, *catA*, and *sigR*p2, respectively. Average fold induction values with standard deviations were obtained from three to five independent experiments.

We previously determined whether various natural or synthetic RACs, regardless of superoxide production, are sensed by the [2Fe–2S]-based regulator SoxR in *S. coelicolor* (Singh et al., 2013). To extend that analysis, we investigated whether these chemicals affect CatR and RsrA, which sense peroxide and thiol-reactive compounds, respectively, by quantifying transcripts from their direct target genes. **Figure 1** shows the responsiveness of SoxR, CatR, and RsrA to actinorhodin (Act, 200 nM), pyocyanin (Pyo, 10 μM), toxoflavin (Tox, 20 μM), phenazine methosulfate (PMS, 50 μM), methyl viologen (paraquat, PQ, 200 μM), plumbagin (PL, 25 μM), menadione bisulfite (MDs, 500 μM), and menadione (MD, 350 μM) at 30 min post-treatment. The results show that each RAC activates the three regulons differentially. Actinorhodin, toxoflavin, and plumbagin effectively activated all three regulons, whereas pyocyanin activated SoxR and CatR/PerR but did not activate RsrA effectively. Paraquat did not activate any of the three regulons, consistent with a previous suggestion that it does not redox cycle to produce ROS in *S. coelicolor*, even though it is redox active to activate *E. coli* SoxR in *S. coelicolor* cells (Singh et al., 2013). On the other hand, menadione bisulfite did not activate SoxR, but activated the CatR regulon, suggestive of redox-cycling activity in *S. coelicolor*. Phenazine methosulfate effectively induced SoxR and CatR target genes, and RsrA-SigR to a lesser extent, similar to the effects of pyocyanin. The induction profiles by these compounds were snapshots obtained 30 min after treatment. Therefore, it is not certain whether the modulation of activity occurred immediately or in a delayed fashion through indirect effects. To resolve this issue, we chose representative RACs and monitored the time courses of their effects on the three regulators.

**FIGURE 2:**
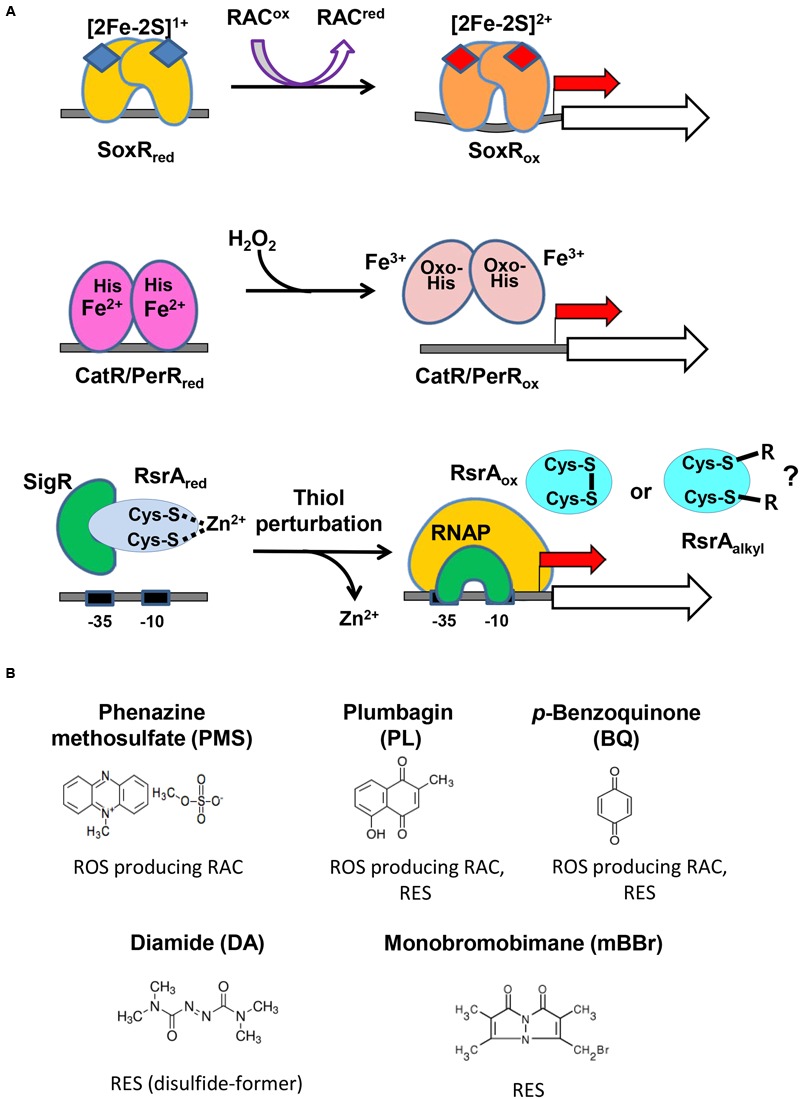
**The redox-sensing schemes of three regulators and the redox-active compounds examined in this study. (A)** The active site metals and amino acid residues in the three sensor-regulators are shown, along with the compounds they react to and the changes they undergo. The redox state of [2Fe–2S] in SoxR changes upon interacting with RACs. The peroxide-sensing Fe^2+^ in CatR/PerR undergo Fenton reaction with H_2_O_2_ to produce hydroxyl radical, and the nearby His is oxidized to oxo-His. Thiol-perturbation by RES cause disulfide bond formation in RsrA to release SigR to transcribe its target genes. Whether RES alkylates RsrA has not been reported. **(B)** Redox-active compounds examined in this study. It is known that phenazine methosulfate (PMS) is an ROS-producer, plumbagin and *p*-benzoquinone are both ROS-producers and reactive electrophile species (RES), and that diamide and monobromobimane are RES.

**Figure 2A** illustrates the activation mechanism of each transcriptional regulator. The [2Fe–2S]^1+^ cluster of SoxR in each monomeric unit is oxidized directly by RAC to [2Fe–2S]^2+^, which changes the binding pattern of SoxR to the promoter region, to activate open complex formation between RNA polymerase and the promoter (Watanabe et al., 2008; Singh et al., 2013; Lee et al., 2015). PerR senses H_2_O_2_ via bound Fe^2+^, which elicits Fenton reaction and oxidation of nearby histidine residues, subsequently leading to the loss of DNA-binding ability of PerR (Lee and Helmann, 2006; Parent et al., 2013). The five amino acids that are critical to sense H_2_O_2_ in PerR are all conserved in *S. coelicolor* CatR, supporting a similar sensing mechanism (Supplementary Figure S1). The reduced RsrA bound to SigR is oxidized via disulfide bond formation between Zn^2+^-coordinating cysteine residues by thiol- RES directly (diamide) or indirectly by depleting MSH via S-conjugation (Kang et al., 1999; Park and Roe, 2008; Rajasekar et al., 2016). Whether RES can inactivate RsrA by direct alkylation has not been reported, even though plausible.

**Figure 2B** shows the five compounds whose effects on the three regulators we monitored *in vivo*. PMS is a known redox-cycling RAC that can produce superoxide radicals and hence peroxides (Nishikimi et al., 1972). Plumbagin and BQ are known to be a redox-cycling RAC and RES (O’Brien, 1991; Castro et al., 2008). Diamide and monobromobimane are electrophiles with different propensities for reaction products. Whereas, diamide rapidly forms disulfide bonds between neighboring cysteine thiols (Kosower and Kosower, 1995), monobromobimane preferentially forms S-alkylated products on cysteine thiols (Kosower et al., 1979). We treated exponentially grown *S. coelicolor* cells with each compound for 5, 10, 20, 40, 60, 80, or 120 min, and measured by S1 nuclease mapping the amount of transcripts from SCO2478, *catA*, and *sigR* promoters that were regulated by SoxR, CatR, and RsrA-SigR, respectively.

### Redox Signaling of PMS in *S. coelicolor*

**FIGURE 3:**
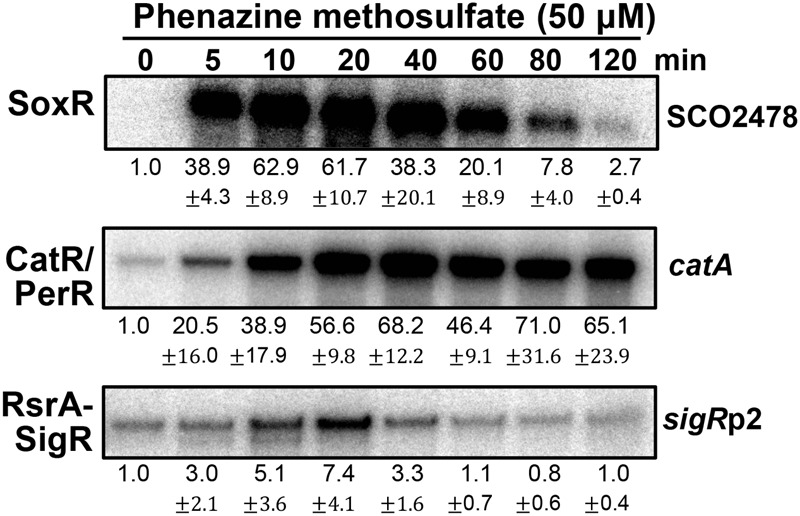
**Effects of phenazine methosulfate, known as a redox-cycling RAC, on the SoxR, CatR/PerR, and RsrA-SigR systems.** Transcripts from the target genes of SoxR (SCO2478), SigR (*sigR*p2), and CatR/PerR (*catA*) were monitored by S1 nuclease mapping. *S. coelicolor* M145 cells were treated with phenazine methosulfate (50 μM) for 5–120 min, prior to cell lysis, to prepare RNA samples. Quantified results from three independent experiments were presented as average values of fold induction with standard deviations.

**Figure 3** shows the effects of PMS on the activities of three regulators at sub-inhibitory doses (Lee et al., 2015). PMS strongly activated SoxR within 5 min, consistent with its direct oxidation of [2Fe–2S]. PMS also inactivated the CatR/PerR repressor (to induce *catA* expression) in less than 5 min, suggesting that it can generate peroxide immediately after treatment. In contrast to the SoxR activation profile, the induction of CatR-regulated *catA* steadily increased for 40 min and then was sustained until 2 h. A previous study showed that the induction of *catA* by 0.2 mM H_2_O_2_ lasted only about 40 min, indicative of the limited duration of H_2_O_2_ following catalase induction (Hahn et al., 2000). Therefore, continuous generation of peroxides by PMS may serve to retain some level of intracellular peroxides to maintain CatR in an inactive form for up to 2 h. The transient mode of SoxR activation by PMS suggests that PMS may be removed from the cell by 2 h post-treatment, likely because of export by one of the target gene products of SoxR (Dela Cruz et al., 2010; Shin et al., 2011). Induction of the SigR-target gene by PMS through oxidizing RsrA appeared quite inefficient. This suggests that the amount of peroxides formed by PMS is not sufficient for immediate disulfide bond formation in RsrA, considering that RsrA is relatively insensitive to hydrogen peroxide (Kang et al., 1999; Rajasekar et al., 2016).

### Redox Signaling of Benzoquinone and Plumbagin

**FIGURE 4:**
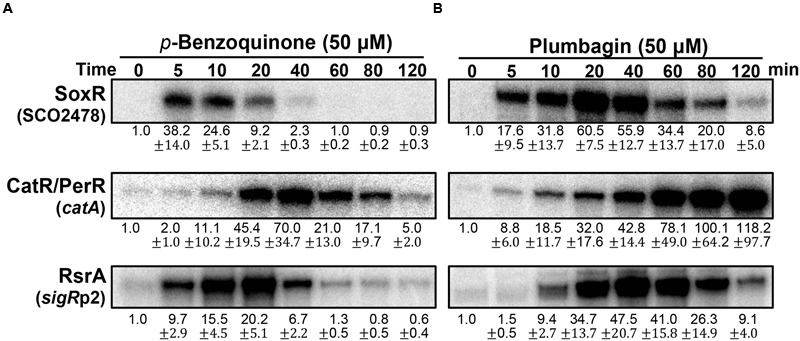
**Effects of *p*-benzoquinone and plumbagin, known as ROS-producing RAC and RES.** *S. coelicolor* cells were treated with either *p*-benzoquinone (50 μM; **A**) or plumbagin **(B)** for 5–120 min. S1 mapping analysis and quantification of results from three independent experiments was performed as in **Figure 3**.

We then examined and compared the action modes of a benzoquinone and naphthoquinone plumbagin at sub-inhibitory doses (Supplementary Figure S2; Lee et al., 2015). As demonstrated in **Figure 4A**, BQ immediately activated the SoxR and SigR systems. In contrast, it induced the CatR system in a delayed fashion. This indicates that BQ is a potent RAC that immediately oxidizes [2Fe–2S] of SoxR, as well as a potent electrophile that can modify or form disulfide bonds in RsrA. Its ROS-producing activity seemed to either be relatively slow or occur through an indirect path via damaging intracellular redox homeostasis. The short duration of both SoxR and SigR activation by BQ suggests that it may be rapidly removed from the cell, possibly by the combined actions of an increased exporter (SoxR target) and mycothiol-mediated detoxification system (SigR target) (Antelmann et al., 2008; Park and Roe, 2008).

The effects of plumbagin are different from those of BQ. Plumbagin induces the SoxR and CatR systems immediately, whereas the SigR system is activated in a delayed fashion (**Figure 4B**). The activation profile of SoxR indicates that plumbagin is a potent RAC, which appears to be removed from the cell more slowly than BQ. Immediate induction of the CatR system suggests that plumbagin is an effective redox-cycling agent in *S. coelicolor.* The continued inactivation of CatR at later time points (60–120 min), when plumbagin is being removed, suggests that either the peroxide level is maintained to keep CatR inactive and/or that oxidized CatR is not rapidly replaced with the functional repressor CatR. In *B. subtilis*, the CatR ortholog PerR is known to be irreversibly oxidized and subject to degradation by LonA (Ahn and Baker, 2016). The delayed induction of the SigR target gene suggests that plumbagin may slowly shift redox homeostasis to favor disulfide formation in RsrA as an indirect consequence, rather than causing oxidation or modifying RsrA directly. Depletion of reduced MSH through S-alkylation could cause a shift in thiol redox homeostasis to favor disulfide formation in RsrA. Shutting off SigR induction at late time points suggests that thiol redox homeostasis is regained within 2 h, most likely because of the restoration of MSH and reduction of protein thiols through the action of SigR target gene products (Park and Roe, 2008; Kim et al., 2012).

### Redox Signaling of Diamide and Monobromobimane in *S. coelicolor*

**FIGURE 5:**
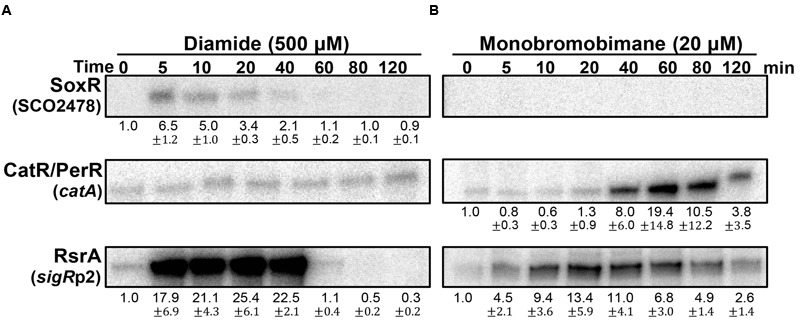
**Effects of diamide and monobromobimane, known as thiol-reactive electrophiles.** *S. coelicolor* cells were treated with diamide (500 μM; **A**) or monobromobimane (20 μM; **B**) for 5–120 min, prior to RNA sample preparation. Results from three independent experiments were presented as described in **Figure 3**.

The redox-signaling activities of diamide and monobromobimane (mBBr), typically known as disulfide-forming and S-alkylating electrophiles, respectively, were then examined at sub-inhibitory concentrations (Supplementary Figure S2). **Figure 5A** shows that diamide is a weak RAC at 0.5 mM, activating SoxR only marginally. It is, however, a potent activator of the SigR system, as previously observed. It did not induce the CatR system, indicating that it does not have any detectable redox-cycling activity. In contrast to diamide, mBBr did not activate SoxR (**Figure 5B**). It moderately activated the SigR system in an immediate and prolonged manner. This agrees with the suggestion that mBBr activates the SigR system by depleting the MSH pool for relatively long time (up to 80 min) at 20 μM, which facilitates disulfide formation in RsrA (Park and Roe, 2008). Interestingly, mBBr induced the CatR system at late time points (**Figure 5B**). This suggests that prolonged depletion of MSH by mBBr resulted in the accumulation of peroxides. In contrast, diamide exerted direct effects on RsrA through immediate disulfide bond formation. The induced SigR system turned off completely within an hour because of disulfide reduction of RsrA by thioredoxin systems and MSH (Kang et al., 1999; Park and Roe, 2008), accompanied by the removal of diamide. Among the induced SigR-target gene products, several nitroreductase candidates may function in degrading diamide, as observed for nitroreductases (AzoR1 and AzoR2) in *B. subtilis* (Antelmann et al., 2008).

### Monitoring ROS Generation by Fluorescence in *S. coelicolor*

The ROS-generating ability of the RACs used in this study was examined using the fluorescent probe 2′-7′-dichlorofluorescein diacetate (DCFH_2_-DA) as previously described (Lee et al., 2015). DCFH_2_ that penetrates the cell membrane is oxidized to fluorescent DCF by ROS and radicals (Winterbourn, 2014). Supplementary Figure S3 shows that only plumbagin and PMS generated detectable fluorescence 30 min post-treatment. These two chemicals induced the CatR system effectively (**Figures 3 and 4B**), consistent with the intracellular oxidation of DCFH_2_. However, the ability of BQ to induce the CatR system (**Figure 4A**) was not revealed by the fluorescence detection assay. Considering that BQ is a good superoxide producer with a one-electron redox potential of +99 mV (O’Brien, 1991) and is likely to produce peroxides, as supported by CatR induction, it appears that the fluorescence assay is not a sufficiently sensitive assay to monitor the ROS-generating ability of RACs in cells.

### Effects of Thiol-Reactive Compounds on RsrA

**FIGURE 6:**
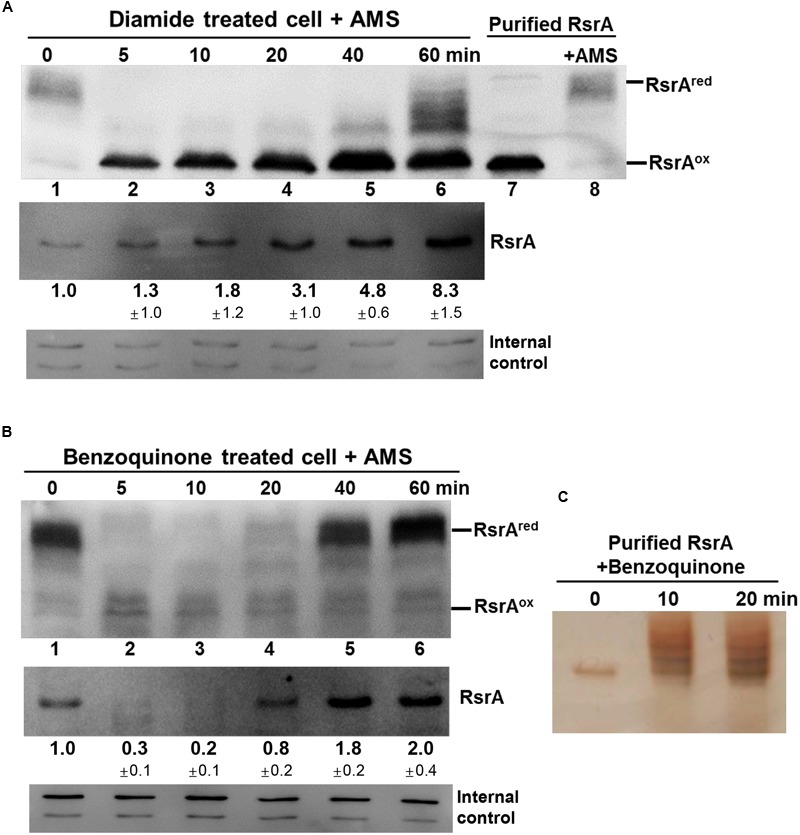
**Effects of thiol-reactive compounds on RsrA.** Effects of diamide and benzoquinone on the redox and modification status of RsrA were examined *in vivo* and *in vitro*. **(A)** Cells were treated with diamide (0.5 mM) for 5–60 min, followed by TCA precipitation and AMS modification of free thiols. Protein samples were resolved by SDS-PAGE, followed by Western blot analysis with anti-RsrA antibody (lanes 1–6). Purified reduced RsrA was either untreated or treated with AMS and run on the same gel as redox standards (lanes 7, 8). To determine the level of total RsrA protein in each sample, extracts from cells prior to TCA precipitation were analyzed in parallel by Western blot analysis (lower panel). The amount of RsrA protein relative to that in the untreated sample was determined from three independent experiments. The non-specific bands were shown at the bottom as internal controls. **(B)** Cells were treated with 50 μM benzoquinone for 5–60 min and analyzed in the same way as in **(A)**. The amount of RsrA was determined from four independent experiments. **(C)** Purified RsrA was treated with 50 μM benzoquinone for 10 or 20 min and analyzed by SDS-PAGE followed by silver staining.

Until now, inactivation of RsrA (or activation of SigR) has been shown to occur via disulfide bond formation, either directly by diamide or indirectly through MSH depletion by the S-alkylating electrophiles *N*-ethyl maleimide (NEM) and mBBr (Park and Roe, 2008; Rajasekar et al., 2016). Slow induction of the SigR system by plumbagin may be caused by a thiol redox shift due to MSH depletion (**Figure 4B**). The rapid and highly potent induction of the SigR system by BQ suggests its direct action on RsrA (**Figure 4A**). We therefore investigated the redox and modification status of RsrA *in vivo* following benzoquinone treatment, in comparison with that following diamide treatment. Immediately after treatment, the TCA-precipitated proteins were treated with AMS to alkylate reduced thiols, followed by Western blot analysis with polyclonal antibody against RsrA. One AMS adduct per thiol adds about 0.5 kDa to the molecular weight of each protein and shifts protein mobility accordingly. For reduced RsrA with seven cysteine residues, up to seven AMS adducts can be formed theoretically. To provide mobility standards for the oxidized vs. reduced RsrA, purified RsrA, either non-treated or treated with AMS, were examined in parallel by SDS-PAGE (Supplementary Figure S4). The multiplicity of AMS-conjugated proteins caused less clear detection due to band broadening in Western blots than the unmodified proteins. The diamide-oxidized RsrA did not interact with AMS, suggesting that the oxidized RsrA with only one or two disulfide bonds hindered AMS to modify the remaining free cysteine thiols. This coincides with a recent finding that the oxidized RsrA maintains the more packed structure than the reduced one (Rajasekar et al., 2016). Results shown in **Figure 6A** indicate that nearly all RsrA in the cell is in the reduced form (**Figure 6A**, lane 1). Upon diamide treatment, RsrA became oxidized within 5 min (lane 2). Reduced forms of RsrA appeared after 40 min and further increased at 60 min. The amount of total RsrA protein in cell extracts before the addition of TCA and AMS was estimated in a separate Western blot analysis (**Figure 6A**, lower panel). As expected, the amount of RsrA steadily increased up to eightfold at 60 min, consistent with the increase in *sigR-rsrA* transcripts by activated SigR (Paget et al., 1998; Kim et al., 2012).

Treatment with BQ produced an unexpected result. Immediately after treatment, the reduced RsrA band disappeared, accompanied with the appearance of a faint oxidized band (**Figure 6B**). The reduced band re-appeared at 40 min post-treatment. When the amount of RsrA in cell extracts for each sample was assessed by Western blot (**Figure 6B**, lower panel), we found that the level of RsrA decreased to ∼30% that of the untreated level at 5 min (lane 2). The protein level then increased steadily after 10 min to about twofold level at 60 min (lanes 3–6). The disappearance of RsrA-specific band can be postulated to be due to degradation and/or inefficient immune-detection of BQ-modified RsrA. Whether benzoquinone can form adducts on RsrA was examined by incubating 50 μM BQ with purified RsrA *in vitro*, followed by SDS-PAGE. Silver staining of the gel showed that BQ caused formation of higher molecular weight forms of RsrA (**Figure 6C**). We analyzed BQ-treated RsrA by LC-MS/MS, which revealed that BQ formed adducts in at least 5 cysteine positions in RsrA (**Table 1**; Supplementary Figure S5; Supplementary Table S1). Therefore, it is very likely that BQ alkylates RsrA *in vivo*. Regardless of whether RsrA is oxidized or modified, the liberated SigR induces its target genes. As suggested by the results shown in **Figure 4A**, nearly all BQ is likely to be removed from cells by approximately 40 min. The time course of the increase in the amount of RsrA and restoration of the reduced form of RsrA by about 40 min coincides with the induction profile of the *sigR*p2 promoter *in vivo* (**Figure 4A**). Therefore, the observed BQ effect reveals a new induction mechanism of SigR regulon by direct modification of RsrA. The antibody detected BQ-conjugated RsrA with significantly lesser efficiency than the unmodified form. Therefore, the fate of BQ-conjugated RsrA requires further systematic investigation.

**Table 1:** Detection of benzoquinone-conjugated cysteines in RsrA by LC–MS/MS.

Cysteine position in RsrA	C3	C11	C31	C41	C44	C61	C62
Frequency of detection^a^	ND^b^	ND	3	2	4	14	3

*^a^The number of times that the mass of the specific cysteine was detected to increase by 108.021 Da, with the SpecE value less than 1E-10. The raw dada for various peptides that contain modified cysteine residues was presented in the Supplementary Table S1 and Supplementary Figure S5.*

*^b^ND^∗^, non-detected.*

**FIGURE 7:**
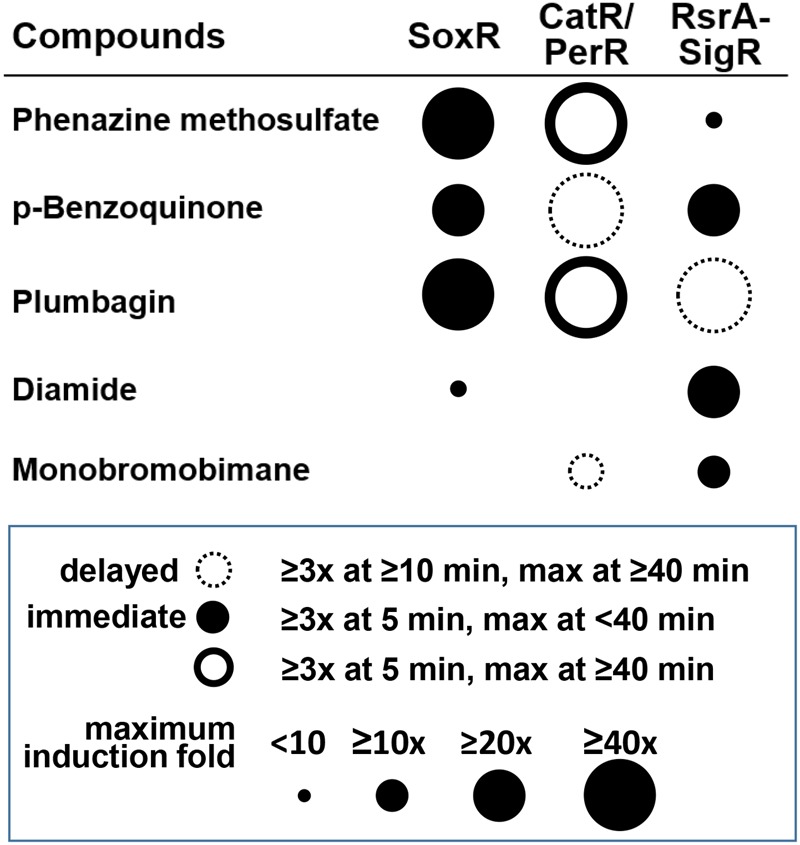
**Summary of the differential signaling effects of reactive compounds on SoxR, CatR/PerR, and RsrA.** For each compound, the effectiveness in modulating the activity of each regulator was presented in terms of fold induction of the target gene (circle size) and the immediacy of the response (filled and empty circles for immediate and delayed responses, respectively). Induction (≥3-fold) at 5 min, with a maximum reached by 40 min, was regarded as an immediate response. Induction at ≥10 min, with a maximum reached by ≥40 min was regarded as a delayed response. PMS and plumbagin caused prolonged CatR response (maximum at ≥40 min), with rapid induction at 5 min, which was represented by a solid-lined open circle.

**Figure 7** summarizes the differential effects of each compound on the three regulators. The results indicate that the differential activities of RACs in cells are well-resolved by examining multiple regulators. The timing and magnitude of their actions and the kinds of regulators that they affect provide information on the possible chemical activity of each of these compounds in cells. Because the induced target genes usually encode proteins with defense functions against the damaging effect of reactive compounds at high doses, this analysis provides insight into the mechanisms of chemical toxicity of the compounds in cells. Responsive regulators in *S. coelicolor* cells unfolded some new activities and signaling pathways of these compounds. Further systematic studies are in need to reveal the life spans of these compounds and the precise mechanisms associated with their secondary effects.

## Author Contributions

J-HR, K-LL, and J-SY conceived the project. J-SY, G-SO, K-LL, and AS performed the experiments. K-LL and J-HR wrote the manuscript.

## Conflict of Interest Statement

The authors declare that the research was conducted in the absence of any commercial or financial relationships that could be construed as a potential conflict of interest.
