# Supplementary material for: Simultaneous Activation of Iron- and Thiol-Based Sensor-Regulator Systems by Redox-Active Compounds
Source: Front Microbiol. 2017 Feb 2;8:139. doi: 10.3389/fmicb.2017.00139 (PMC5288332; doi:10.3389/fmicb.2017.00139)
Supplement: Supplementary file 2 [file Data_Sheet_1.DOCX]

**Supplementary Information**

**Simultaneous activation of iron- and thiol-based sensor-regulator systems by redox-active compounds**

Kang-Lok Lee^†^, Ji-Sun Yoo^†^, Gyeong-Seok Oh, Atul K. Singh, and Jung-Hye Roe^*^

School of Biological Sciences and Institute of Microbiology, Seoul National University, Seoul 151-742, Korea

†Kang-Lok Lee and Ji-Sun Yoo contributed equally to this work.

***Correspondence**:

Jung-Hye Roe

[jhroe@snu.ac.kr](mailto:jhroe@snu.ac.kr)


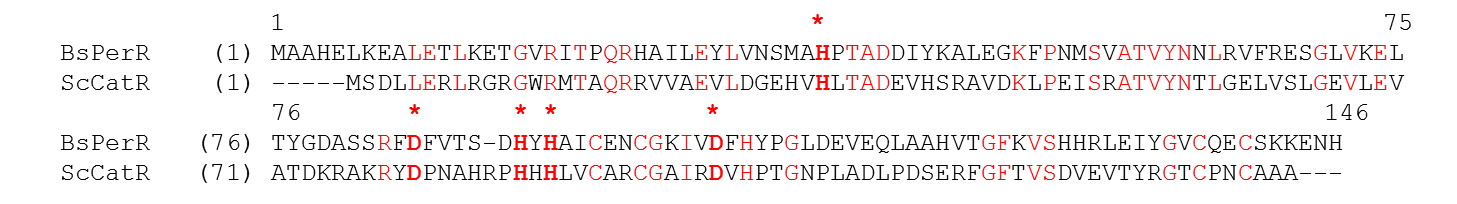


**Figure S1.** Comparison of *B. subtilis* PerR and *S. coelicolor* CatR. The residues of BsPerR that may coordinate the regulatory iron and known to be critical for sensing peoxides (Lee *et al*., 2006) are marked by asterisk (*). They are conserved in ScCatR.


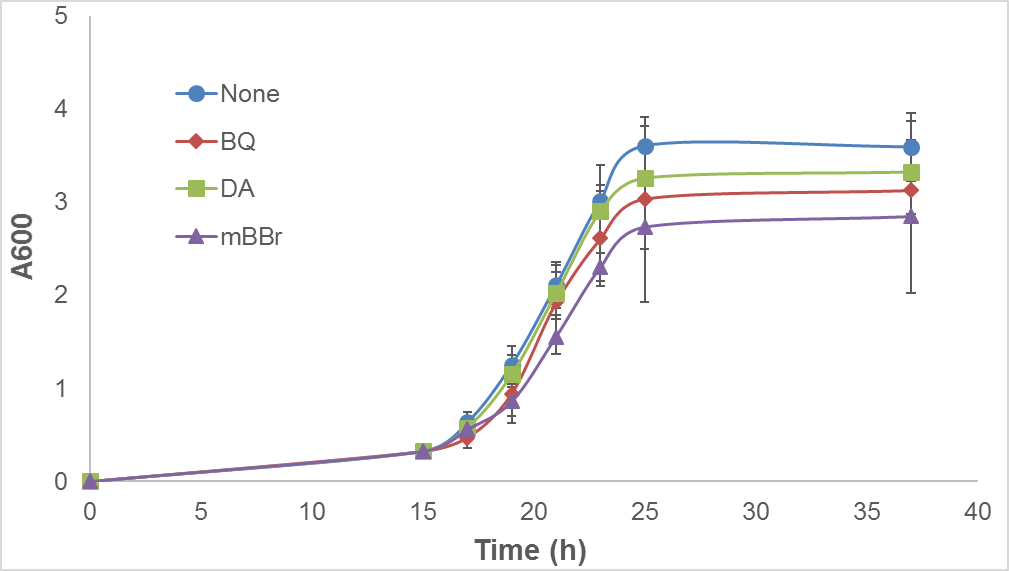


**Figure S2.** Effect of various RACs on the growth of *S. coelicolor.* Wild-type *S. coelicolor* (M145) cells were grown in YEME liquid medium by inoculating 10^8^ spores per 100 ml medium in 1 L flask, until OD600 reaches 0.2–0.3 (mid exponential phase) after 15 h incubation by shaking at 30°C. Chemicals at the indicated final concentrations used in this study were then added: para-benzoquinone (BQ; 50 μM), diamide (DA; 500 μM), or monobromobimane (mBBr; 20 μM). Cell growth was subsequently monitored by measuring OD600. Growth of non-treated cells was monitored in parallel. At least three independent experiments were performed for each compound, to present average values with standard deviations


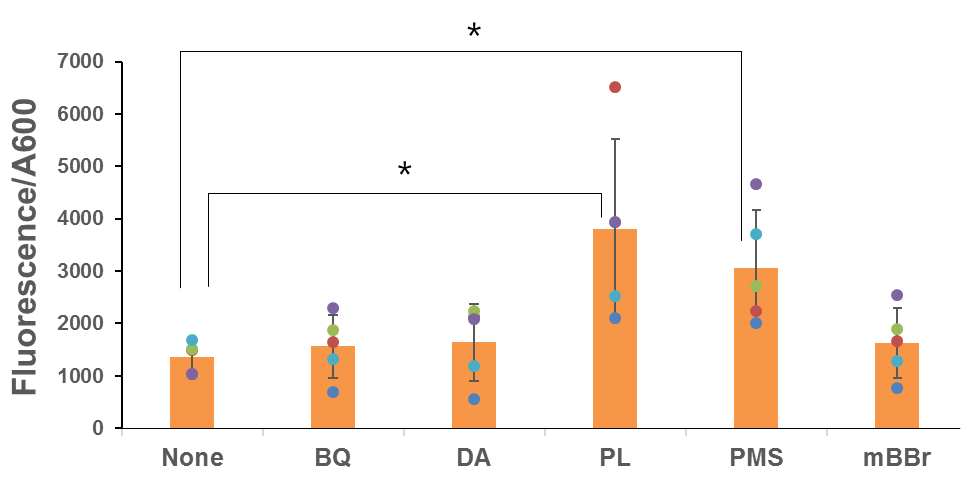


**Figure S3**. Fluorescence-mediated ROS detection in *S. coelicolor* cells. Exponentially grown *S. coelicolor* cells (OD600 ~ 0.2 to 0.3) were treated with para-benzoquinone (BQ, 50 µM), diamide (DA, 500 µM), plumbagin (PL, 50 µM), phenazine methosulfate (PMS, 50 µM), or monobromobimane (mBBr, 20 µM) for 30 min. Accumulated peroxides in the cell were detected by peroxide-reactive fluorescence probe, 2′-7′-dichlorodihydrofluorescein diacetate (DCFH-DA), with excitation at 492 nm and emission at 535 nm. Bar graph and color of each dot means average values and data acquired from independent experiment, respectively. Five different experiments were done to obtain average values with standard deviations. Asterisks (*) indicate measurements with P-values of less than 0.05 by Student’s t-test.


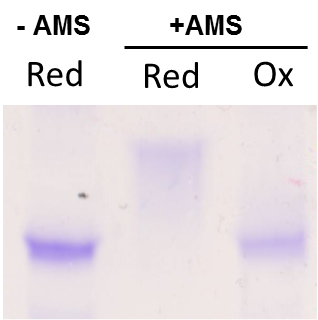


**Figure S4**. AMS modification of diamide-oxidized and reduced RsrA. Reduced RsrA was prepared by incubating 200 nM RsrA in 10 mM DTT at 25°C for 1 h with subsequent addition of 400 nM ZnSO_4_ for 1 h at 5% H_2_, 5% CO_2_ and 90% N_2_ in the anaerobic chamber (Coy). Free zinc and DTT was removed by PD-10 desalting column using anaerobically prepared TN buffer. The reduced RsrA was oxidized by 0.5 mM diamide for 30 min. The reduced or oxidized RsrA was treated with 0.5 mM 4-acetamido-4'-maleimidylstilbene-2,2'-disulfonic acid (AMS) for 20 min, and analyzed by SDS-PAGE, followed by Coomassie blue staining.


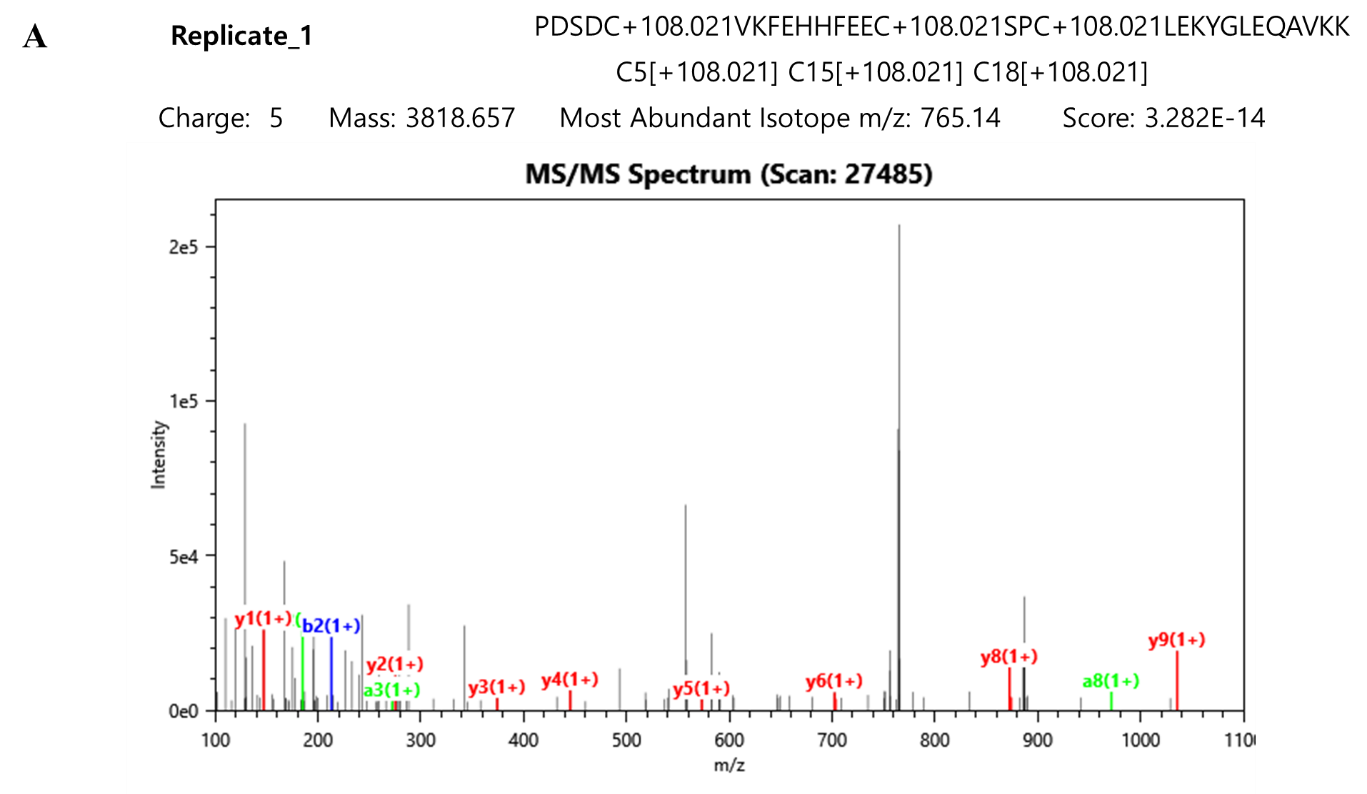


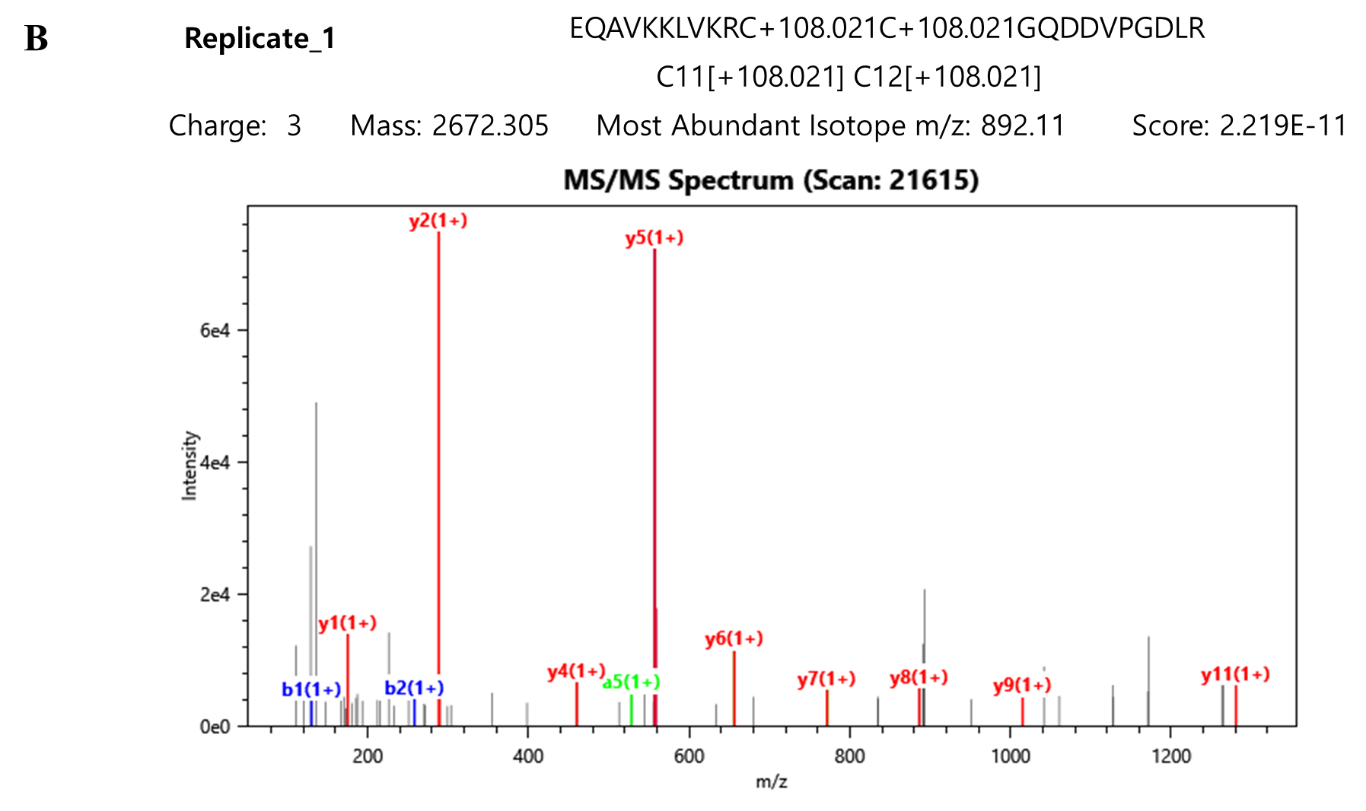


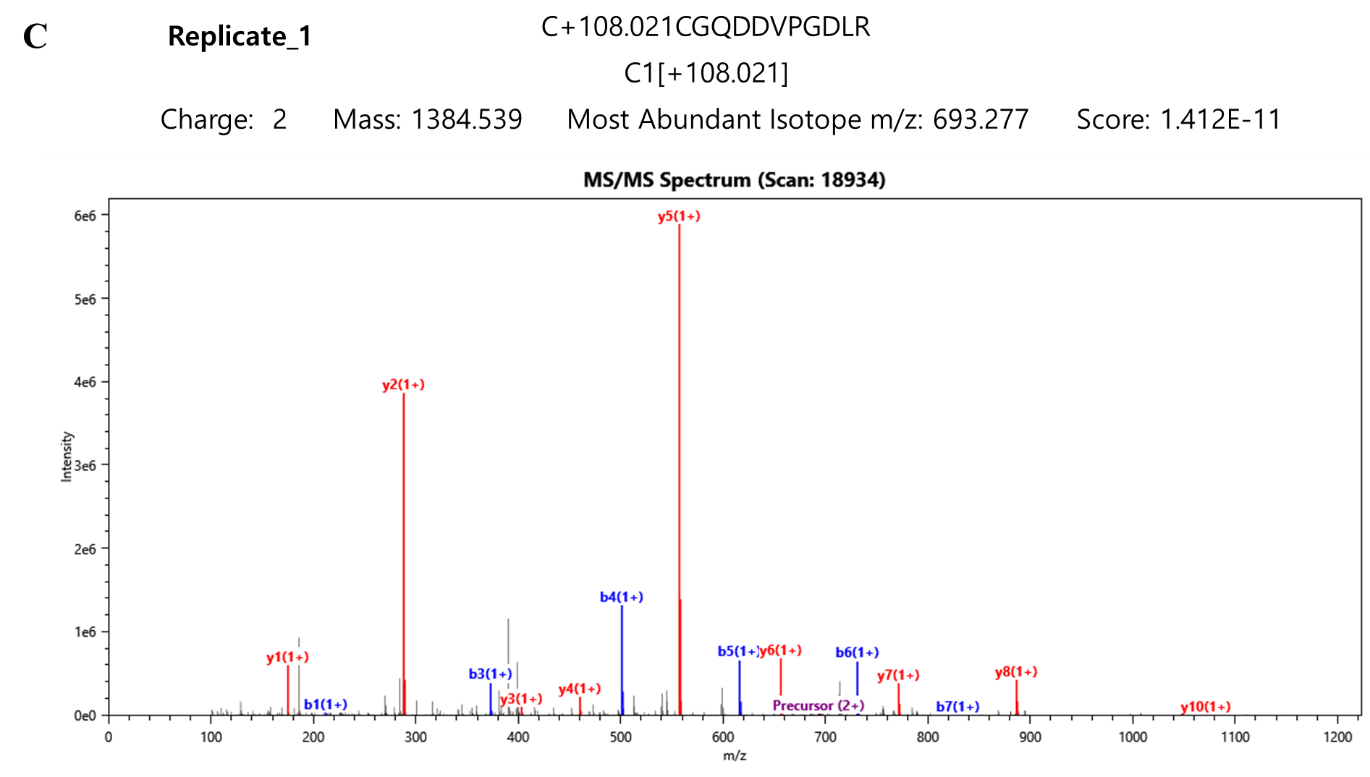


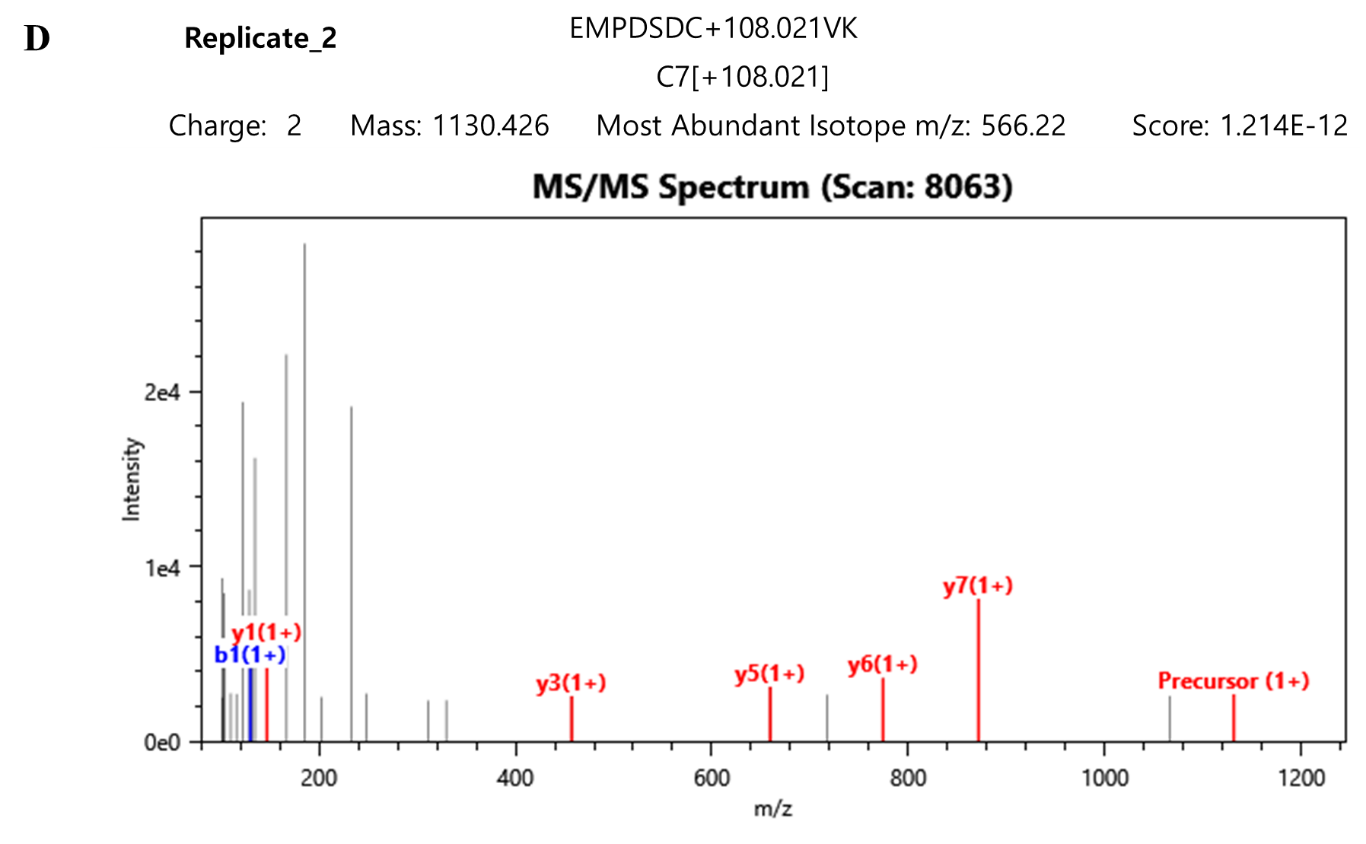


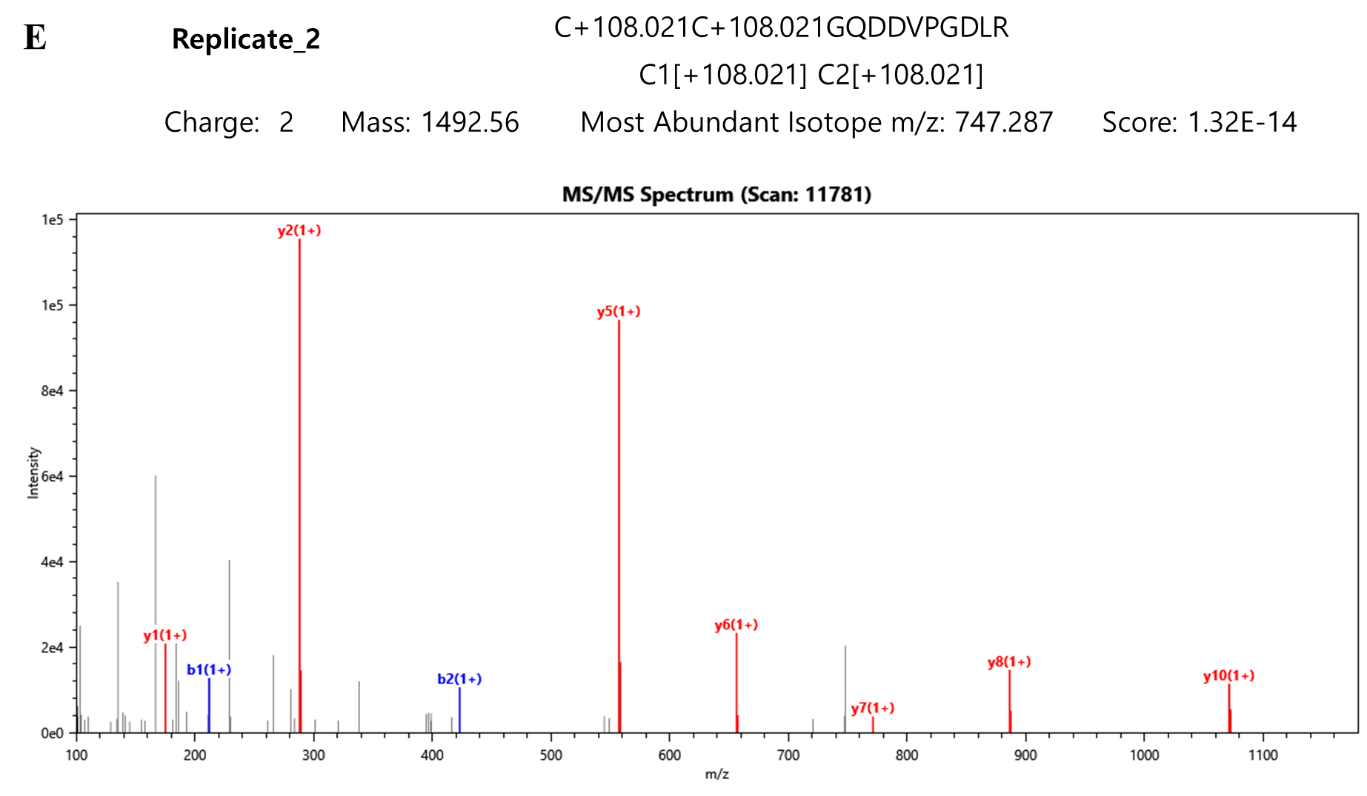


**Figure S5**. MS/MS spectra of benzoquinone-modified RsrA peptides. 50 µM benzoquinone-modified RsrA in 300 µl buffer was denaturated using 8 M urea for 1 h at 37℃ and reduced with 10 mM DTT for 1 h at 37℃. The reduced samples were diluted with 6 volumes of 50 mM ammonium bicarbonate buffer (pH 7.8) with final 1 mM CaCl_2_. Tryptic digestion was performed for 3 hours at 37˚C with 1:50 (w/w) trypsin-to-protein ratio, followed by C-18 SPE clean up. Digested peptides were analyzed on an UltiMate 3000 R and Q Exactive Benchtop LC-MS/MS (Thermo Scientific). All MS/MS data were searched by MS-GF+ algorithm. Panels A, B, C, D, E show MS/MS spectra of peptides containing residues 27 – 56, 51 – 72, 61 – 72, 25 – 33, and 61 – 72 of benzoquinone-modified RsrA, respectively
